# The *Caenorhabditis elegans* gene *ham-1* regulates daughter cell size asymmetry primarily in divisions that produce a small anterior daughter cell

**DOI:** 10.1371/journal.pone.0195855

**Published:** 2018-04-18

**Authors:** Jerome Teuliere, Ismar Kovacevic, Zhirong Bao, Gian Garriga

**Affiliations:** 1 Department of Molecular and Cell Biology, University of California Berkeley, Berkeley, California, United States of America; 2 Developmental Biology Program, Sloan Kettering Institute, New York City, New York, United States of America; University of North Carolina at Chapel Hill, UNITED STATES

## Abstract

*C*. *elegans* cell divisions that produce an apoptotic daughter cell exhibit Daughter Cell Size Asymmetry (DCSA), producing a larger surviving daughter cell and a smaller daughter cell fated to die. Genetic screens for mutants with defects in apoptosis identified several genes that are also required for the ability of these divisions to produce daughter cells that differ in size. One of these genes, *ham-1*, encodes a putative transcription factor that regulates a subset of the asymmetric cell divisions that produce an apoptotic daughter cell. In a survey of *C*. *elegans* divisions, we found that *ham-1* mutations affect primarily anterior/posterior divisions that produce a small anterior daughter cell. The affected divisions include those that generate an apoptotic cell as well as those that generate two surviving cells. Our findings suggest that HAM-1 primarily promotes DCSA in a certain class of asymmetric divisions.

## Introduction

*Caenorhabditis elegans* somatic development is essentially invariant. Almost all of the somatic divisions are asymmetric, generating two daughter cells that differ in fate [1, 2]. Studies of Asymmetric Cell Division (ACD), primarily in *C*. *elegans* and *Drosophila melanogaster*, have led to insights into the molecules and mechanisms that distribute developmental potential to the two daughter cells in an ACD. Many of these molecules control not only the distribution of fate but also the orientation of the mitotic spindle, a process that is critical for the appropriate distribution of fate unequally to daughter cells [3–5].

Some of these ACDs are also asymmetric in furrow positioning, resulting in daughter cells of different sizes. The stem cell divisions of Drosophila neuroblasts, for example, exhibit Daughter Cell Size Asymmetry (DCSA) to produce a larger neuroblast and a smaller Ganglion Mother Cell (GMC) or Intermediate Progenitor (INP), cells with more limited developmental potential [3]. Other examples of DCSA are *C*. *elegans* neuroblast (NB) divisions that produce an apoptotic daughter cell. These divisions produce a larger cell that either differentiates into a neuron or will divide again (abbreviated S for Survival) and a smaller cell that dies (abbreviated x) [6–9]. The majority of these ACDs are oriented along the anterior posterior (AP) axis and thus can be classified either as a(x)P(S)-type (small anterior cell that dies-x/LARGE Posterior cell that survives-S) or as A(S)p(x)-type (LARGE Anterior cell that survives-S/small posterior cell that dies-x) in this study. These NB divisions require several molecules that appear to be dispensable for divisions that do not exhibit DCSA [10].

One surprise is that DCSA in NB divisions that produce an apoptotic cell can result from at least two distinct mechanisms in *C*. *elegans*. This distinction was made in Q.a and Q.p, the anterior and posterior daughters, respectively, of the Q neuroblast. The Q.a ACD is an a(x)P(S)-type that produces a smaller daughter cell that dies, Q.aa. A spindle-independent, myosin-dependent mechanism contributes to Q.a DCSA. The Q.p ACD is an A(S)p(x)-type that produces a smaller daughter cell that dies, Q.pp. A spindle-dependent, myosin-independent mechanism contributes to Q.p DCSA [9]. Although Q.a and Q.p divide using distinct mechanisms, both divisions require several of the same molecules for DCSA, including CNT-2, GRP-1 and PIG-1 proteins [6, 9, 11, 12]. The requirement for these genes in Q.a and Q.p DCSA suggest that the divisions share mechanistic features.

HAM-1 differs from other molecules in that it regulates DCSA in Q.a but not Q.p [13, 14]. HAM-1 localizes to the cortex and nucleus [14, 15] and has been reported to be a transcription factor that regulates the DCSA gene *pig-1* [13]. Many but not all divisions that produce an apoptotic daughter require HAM-1. Here, we describe a survey of the cells that require HAM-1 and show that HAM-1 loss primarily affects a(x)P(S)-type NB divisions. We also find that HAM-1 loss also alters DCSA in a(S)P(S)-type divisions that occur with an aP-type polarity but produce two cells that survive. These latter observations suggest that the role of HAM-1 in apoptosis is indirect and a consequence of altered DCSA. We discuss how HAM-1 might function in DCSA.

## Materials and methods

### Genetics

General handling and culture of nematodes were performed as previously described [16]. The N2 Bristol line was used as wild type, and experiments were performed at 20°C unless otherwise noted. The following mutations and integrated arrays were used:

*LG I*. *zdIs5* [*mec-4p*::*gfp*] [17].
*LG II*. *ynIs25* [*flp-12p*::*gfp*] [18, 19].
*LG III*. *nIs107[tbh-1p*::*gfp + lin-15(+)]* [20], *rdvIs1* [*egl-17p*::*mCherry*:*his-24 + egl-17p*::*myristolated mCherry + pRF4*] [9].
*LG IV*. *ham-1(gm279)* [7].
*LG V*. *zuIs178[(his-72p 1kb*::*HIS-72*::*GFP); unc-119(+)]* [21].
*LGX*. *gmIs81*[*mec-4p*::*mCherry*, *flp-12p*::*EBFP2*, *gcy-32p*::*gfp*, *egl-17p*::*gfp*] [22].
Unmapped: *ltIs44[pie-1p-mCherry*::*PH(PLC1delta1) + unc-119(+)]* [23], *leIs2702[ceh-19p*::*gfp; unc-119(+)]* [24], *lqIs80[scmp*::*gfp*::*CaaX]* [25].
Extra-chromosomal arrays: *kyEx581[ocr-4p*::*gfp + lin-15(+)]* (Tobin et al 2002), *Ex [srbc-66p*::*gfp; unc-122p*::*dsRed]* [26].

### Neuron number scoring

All neurons were detected with transcriptional reporters that express fluorescent proteins under control of the indicated *C*. *elegans* promoter. The A/PVM, SDQR/L, A/PQR and URXR/L neurons were detected using the *gmIs81* reporter. The SMB, OLQ, ASK, MC and RIC neurons were detected using the reporters *flp-12p*::*gfp*, *ocr-4p*::*gfp*, *srbc-66p*::*gfp*, *ceh-19p*::*gfp* and *tbh-1p*::*gfp*, respectively. When an extra neuron was detected in a *ham-1* mutant, its position was in close proximity to the normal position of the single neuron found in wild-type animals. Missing neurons were only scored when using integrated transgenes, since extra-chromosomal arrays can be lost during cell divisions. Statistical analysis was performed using the two-sample Z-test for proportions.

### Neuroblasts daughter size measurements

T.p lineage analysis was performed in early L2 larvae using *lqIs80[scmp*::*gfp*::*CaaX]*. V5.pa lineage analysis was performed by compiling and reordering images of *rdvIs1[egl-17p*::*myr mcherry + egl-17p*::*H2B*::*mcherry]* L2 larvae. The mcherry markers are upregulated in all cells of the V5.pa lineage. V5.paa daughter cells size measurements were performed at the 3- and early 4-cell stages, before V5.paap and V5.paaa migrations occurred. T.pp and V5.pa neuroblast daughter cell sizes measurements were performed as previously described for the Q neuroblasts' daughters [11, 12]. The daughter cells sizes of the P cells 3–8 daughter cell sizes were determined using *gmIs81*[*mec-4p*::*mCherry*, *flp-12p*::*EBFP2*, *gcy-32p*::*gfp*, *egl-17p*::*gfp*], which labels the cells of the P3-8 lineage. Ratios are indicated in the text ± standard deviation.

For embryonic neuroblasts, automated lineaging was performed as described [27]. After neuroblast identification, the size asymmetry ratios were determined by dividing the area of the surviving sister by the area of the dying sister as estimated at the division plane immediately following cytokinesis. Two embryos of each genotype were analyzed. Some divisions could not be scored due to ambiguity of the membrane label.

For each genotype and ACD, the measured daughter cells size ratio was plotted on graphs where horizontal dotted lines indicate 1:1 ratios, and horizontal grey bars indicate the median of each ratio distribution. Statistical analysis was performed using the Mann-Whitney U-test.

## Results

### All neuroblast lineages that normally produce anterior daughter cells that die have an abnormal number of neurons in *ham-1* mutants

**Fig 1:**
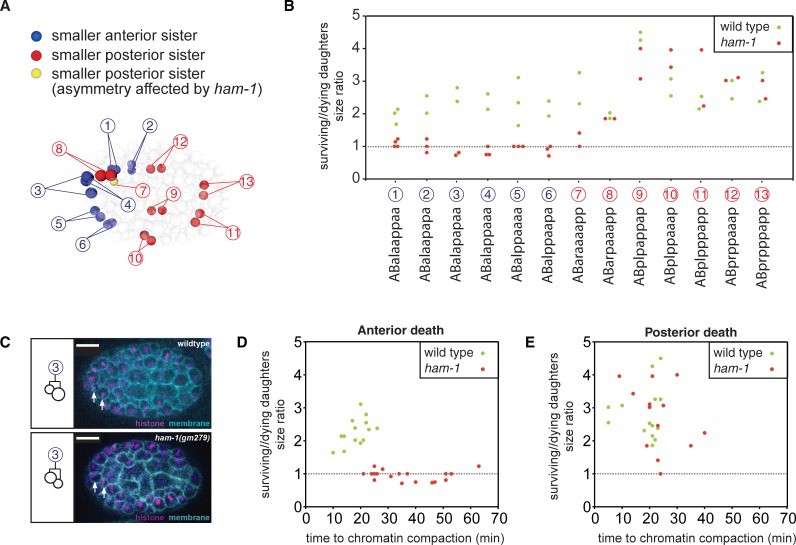
Automated lineaging of 13 neuroblast divisions that produce an apoptotic daughter cell. (A) Schematic representation of an embryo at 265 mins of development showing the positions of the 13 cells (or their daughters) whose divisions were analyzed. Cells are color-coded to describe which ones divide to produce an anterior daughter (blue) or posterior daughter (red) that dies. ABaraaaap (#7), which produces a posterior daughter that dies and whose division asymmetry is affected by *ham-1* loss, is labelled in yellow. (B) Asymmetry ratios of all analyzed daughter cells in wild-type (green circles) and *ham-1(gm279)* (red circles) embryos. The name of the apoptotic cell (e.g., ABalaappaa) is represented along the X-axis with the corresponding number of daughter cells doublet used in (A). (C) A representative division of the ABalapapa cell (#3) yielding an anterior apoptotic daughter in wild-type (upper panel) and *ham-1(gm279)* (lower panel) embryos. Arrows indicate the two daughters, with anterior to the left. In the *ham-1(gm279)* mutant embryos, the asymmetry is reversed and reduced. The plasma membrane is labeled with *pie-1*::*PH*::*mCherry* and the nucleus is visualized with *his-72*::*HIS-72*::*GFP* (REFs). Scale bars: 10 μm. (D, E) Comparison of asymmetry ratios for wild-type and *ham-1(gm279)* embryo to the time of chromatin compaction, a marker for apoptosis.

Previous studies using neuronal specific markers showed that *ham-1* mutants produce abnormal numbers of neurons in specific lineages [7, 13–15, 28]. Further analysis of these studies revealed that most extra cells arise appear to arise from 21 of 34 (32 embryonic and 2 post-embryonic) neuroblast divisions that produce an anterior cell fated to die and a posterior cell that survives and adopts either a neuronal or mitotic fate [1, 2] (Table 1)(Fig 1). The *ham-1* mutant HSN/PHB, ALN/PLM and CEPD/URX lineages are also missing neurons, resulting from either ectopic apoptosis or a failure of the neurons to differentiate and to express the appropriate marker [7, 14, 15, 28, 29].

**Table 1:** *ham-1* mutant extra cell phenotypes.

Apoptotic cell	Lineage name*	Time of death post-fertilzation (min)	Reference for *ham-1* involvement
**ABalppaapa (6)**	CEPsoVL	260	[7], this study
**ABalapapaa (3)**	OLQDL	260	this study
**ABalaappaa (1)**	OLQsoVR/AVAR	260	[7], this study
**ABalaapapa (2)**	CEPsoVR	260	[7], this study
**ABalppaaaa (5)**	OLQsoVL/AVAL	265	[7], this study
**ABalappaaa (4)**	RID	275	[7, 14], this study
ABpraaaappa	ASKR	310	this study
ABalpppappa	ASKL	310	this study
**ABprapppapa**	HSNR/PHBR	315	[7]
**ABplapppapa**	HSNL/PHBL	315	[7]
ABprapapppa	ALNR/PLMR	320	[7]
ABplapapppa	ALNL/PLML	320	[7]
ABplaaaaapa	URXL/CEPDL	330	[28], this study
ABarpapaapa	URXR/CEPDR	330	[28], this study
ABalpappaaa	I2L	330	[7]
ABalpapappa	SMBVL	335	this study
ABprppaapaa	RIMR	340	[7]
ABprapaaaaa	ADAR/ADER	340	[7, 14]
ABplppaapaa	RIML	340	[7]
ABplapaaaaa	ADAL/ADEL	340	[7, 14]
ABarapapaaa	I2R	340	[7]
ABalpaaappa	MCL	370	this study
ABalaapappa	CEPsoVR	385	[7]
ABalaapaapa	ILshR	390	NT
ABalaaaarra	RMER	400	ambiguous [7]
ABalaaaarla	RMEL	400	ambiguous [7]
ABalaaaalpa	ILshL	400	NT
ABalppaappa	CEPsoVL	410	[7]
ABalpapapapa	SMBDL	420	this study
ABarappapapa	SMBDR	425	this study
ABprppaaaapa	RICR	440	this study
ABplppaaaapa	RICL	440	this study
**(ABprappaaa)QR.aa**	QR.a	1280	[13]
**(ABplapapaaa)QL.aa**	QL.a	1300	[13]

The top six (1–6) apoptotic cells are produced by the similarly numbered neuroblast divisions in Fig 1. All somatic neuroblast divisions (32 embryonic and 2 post-embryonic divisions) that produce an anterior apoptotic cell are listed and ordered by time of birth. Cells in bold are known to be larger in *ham-1* mutants.

*The name that we use to describe the lineage. In some cases these are neurons or glia cells produced by the lineage (e.g., CEPsoVL) and in other cases it is the neuroblast (e.g., Q.a).

**Fig 2:**
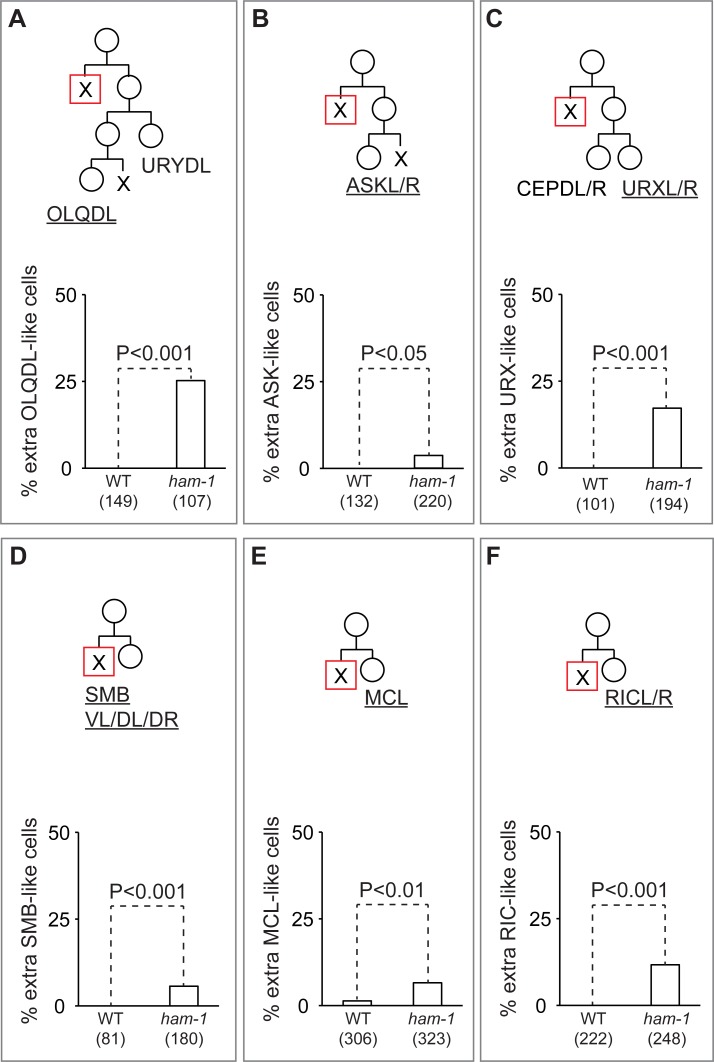
Abnormal numbers of neurons in nine neuroblast lineages that produce an anterior apoptotic daughter cell. (A) OLQDL, (B) ASKL/R, (C) URXL/R, (D) SMBVL/DL/DR, (E) MCL and (F) RICL/R lineage diagrams (upper panels) and the frequency of at least one extra neurons produced in wild-type (WT) and *ham-1* mutant animals (lower panels). For each lineage, the neurons labelled are underlined on the diagram. Cells fated to die are indicated by an x, the anterior dying cell in each lineage is indicated by an x in a red square. The number of animals scored is indicated in parenthesis below each genotype.

To determine whether *ham-1* loss affects other neuroblast lineages, we scored the number of neurons produced by 9 of the 11 remaining embryonic a(x)P(S)-type neuroblast divisions (OLQDL, ASKL/R, SMBVL, MCL, ILshL/R, SMBDL/R, RICL/R). The two lineages that produce the left and right ILsh cells were not analyzed due to the lack of a specific marker. Fluorescent markers for the neurons ASKL/R, RICL/R, MCL, SMBVL, SMBDL/R and OLQDL revealed that *ham-1* mutants produced extra neurons that expressed these markers (Fig 2A, 2B and 2D–2F). We also confirmed the effects of *ham-1* mutations on some of the previously studied *ham-1*-dependent lineages (Table 1; Fig 2C).

The remaining neuroblast divisions that produce apoptotic cells in the *C*. *elegans* hermaphrodite are oriented along the dorsal-ventral (DV) axis, the left-right axis (LR) or are A(S)p(x)-type divisions. The DV-oriented divisions that produce the ADL neurons generate extra neurons in *ham-1* mutants, but the DV-oriented divisions that produce the NSM neurons do not [7, 14]. The neurons of three A(S)p(x)-type divisions have been analyzed, and these lineages failed to produce extra neurons in *ham-1* mutants [7, 13, 28]. We scored the numbers of M4 and PVD neurons, each produced by A(S)p(x)-type divisions, and did not observe extra neurons. Taken together, these findings suggest that *ham-1* primarily regulates neuroblast divisions that produce an anterior daughter that dies. We note, however, that 7 of the 34 divisions that produce an anterior apoptotic cell also generate a posterior cell that dies in a subsequent division (for examples, see the URYDL/OLQDL and ASKL/R lineages in Fig 2A and 2B). Analysis of the URYDL/OLQDL and RID lineages in the next section supports the hypothesis that it is the division that produces the anterior apoptotic daughter cell that is affected by *ham-1* loss.

### Loss of neuroblast daughters size asymmetry in *ham-1* mutants

Due to the challenging nature of directly observing neuroblast divisions, neuron numbers have been the primary phenotype used to assess *ham-1* mutant defects. Two notable exceptions are the direct observations of the embryonic HSN/PHB and larval Q.a divisions [7, 13]. Observing wild-type divisions that produce a dying cell revealed that the surviving cell is larger than its apoptotic sister [6–9], Strikingly, both the HSN/PHB and Q.a neuroblast divisions display a similar *ham-1* mutant phenotype: the daughter-cell-size asymmetry (DCSA) between the two daughter cells is lost or reversed.

Given the effects of the *ham-1* mutations on a(x)P(S)-type neuroblast ACDs, we further tested whether these divisions are associated with DCSA defects by following several embryonic divisions and measuring DCSA using automated cell lineage tracing [27]. We fluorescently labeled embryonic nuclei and plasma membranes to track cell divisions from the four-cell stage onward. Because membrane overlap in deeper optic sections of the embryo precludes the measurement of cell sizes, analysis was restricted to 13 neuroblasts that divide at the surface of the embryo and produce a cell that dies (Fig 1A). All a(x)P(S)-type divisions we could observe (including the great-grandmother of the OLQDL neuron, Fig 1B and 1C) are abnormal in *ham-1* mutants. On average, wild-type divisions that generate an anterior apoptotic cell had a posterior:anterior size ratio of 2.27 (n = 14, SD = 0.41), while the *ham-1* mutant divisions had a ratio of 0.95 (n = 18. SD = 0.16) (P<0.0001; Mann-Whitney U-test). By contrast, the size asymmetry of six of the seven A(S)p(x)-type NB divisions we observed were unaffected by *ham-1* loss. Divisions that generate a posterior apoptotic daughter had an average asymmetry ratio of 2.83 (n = 14, SD = 0.79) in wild-type embryos and of 2.74 (n = 14. SD = 0.97) in *ham-1* mutant embryos (no significant difference; Mann-Whitney U-test) (Fig 1B). These observations confirm that *ham-1* is preferentially required to generate DCSA in a(x)P(S)-type NB divisions.

Automated lineaging also allowed us to estimate the time of apoptosis onset by following chromatin compaction. Although the *ham-1* mutation often altered daughter cell size, it did not alter which daughter cell died. For example, the daughter cell size ratio reversed in three divisions of ABalapapaa, yet the anterior daughter still died in all three cases. The extra neurons produced by many *ham-1* mutant lineages predict that some of these embryonic divisions might produce two daughter cells that fail to die, yet none of the divisions did. This apparent discrepancy can be explained by the low penetrance of extra cells produced by some lineages and the small number of embryos analyzed. Apoptosis onset in *ham-1* mutant anterior daughter cells, however, took up to three times longer than in the same wild-type cells (P<0.0001; Student's t-test) (Fig 1D). By contrast, all posterior deaths occurred with similar timing in wild-type and *ham-1* mutant embryos (no significant difference, Student's t-test) (Fig 1E), including ABaraaaap, which is the only posterior apoptotic cell produced by an altered asymmetric division in the *ham-1* mutant. A similar delayed death phenotype was observed by Wei et al in the enlarged apoptotic daughter of the NSM neuroblast of *pig-1* mutants [30]. In conclusion, our embryonic lineaging suggests that *ham-1* is required for normal DCSA and indirectly for apoptosis in the subset of neuroblast ACDs that produce anterior daughter cells that die.

### *ham-1* regulates DCSA in neuroblast divisions with two surviving daughters

**Fig 3:**
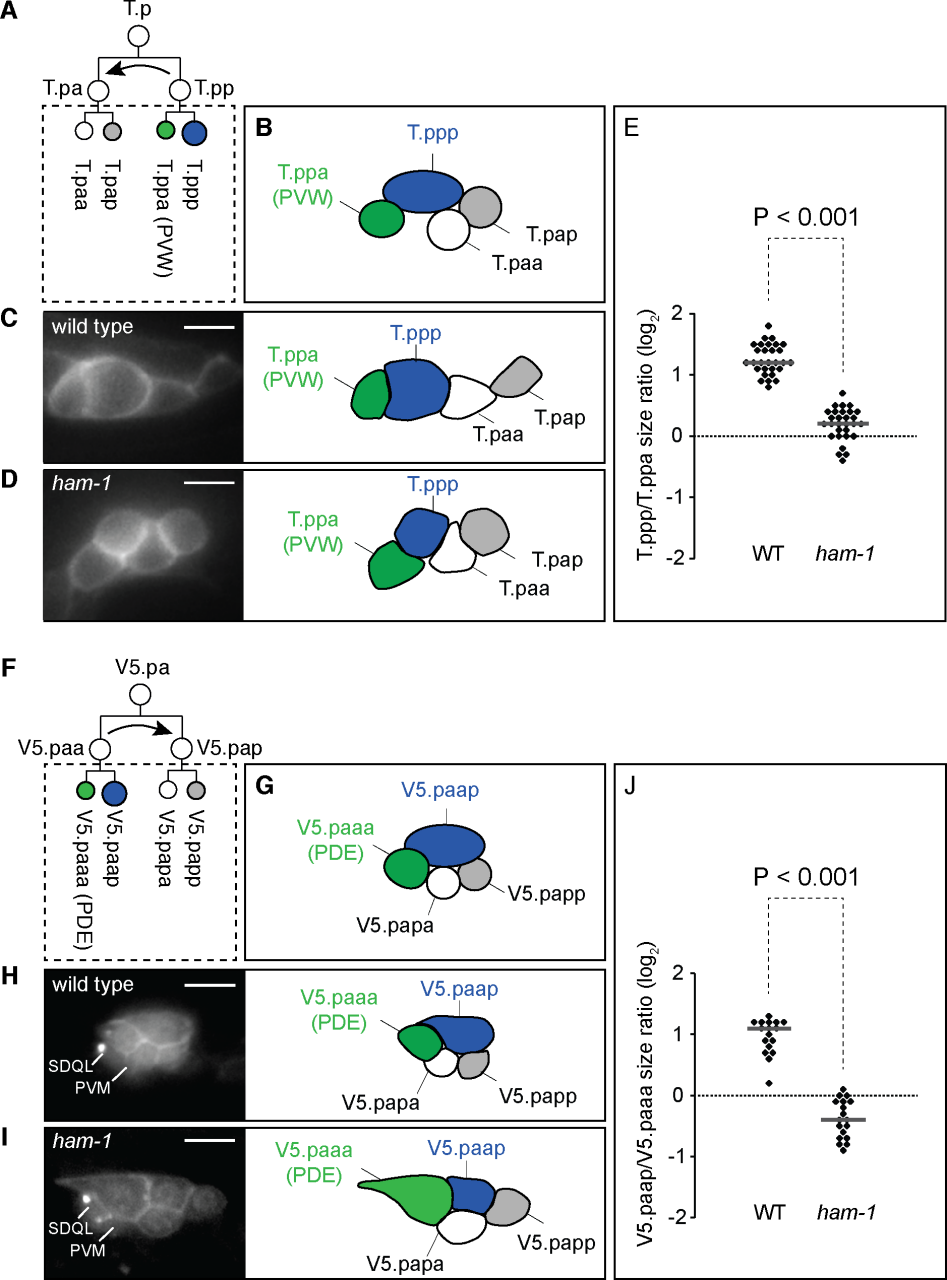
Daughter cells size asymmetry (DCSA) defects in the *ham-1* mutant T.pp and V5.paa divisions. (A) Schematic representation of the divisions in the T.p lineage. The T.pp division produces daughter cells of different fates and sizes, whereas the T.pa division produces daughter cells that only differ in fate. Prior to division, T.pp migrates dorsally and anteriorly to T.pa as indicated by a curved arrow [1]. The dotted square indicates the four cells depicted in B and the right panels of C and D. (B) Schematic representation of the relative positions of the four cells T.ppa/pppp (in green and blue, respectively) and T.paa/pap (in white) in the left T.p lineage. (C-D) Fluorescent labeling of the left T.p lineage at the 4 cells stage by the transgene *lqIs80[scmp*::*gfp*::*CaaX]* (left panels) and schematic representations of the corresponding four cells (right panels) in wild-type (C) and *ham-1(gm279)* (D) larvae. Scale bars: 10 μm. (E) T.ppp/T.ppa cell size ratios in wild-type and *ham-1* mutant animals. (F) Schematic representation of the first two rounds of division in the V5.pa lineage. The V5.paa division produces daughter cells of different fates and sizes, whereas the V5.pap division produces daughter cells that only differ in fate. Prior to division, V.paa migrates dorsally to V5.pap as indicated by the curved arrow. The dotted square indicates the four cells depicted in G and the right panels of H and I. (G) Schematic representation of the relative positions of the four cells V5.paaa/p (in green and blue, respectively) and V5.papa/p (in white) in the left V5.pa lineage. (H-I) Fluorescent labeling of the left V5.pa lineage at the 4 cells stage by the transgene *rdvIs1[egl-17p*::*myristoylated mcherry; egl-17p*::*H2B*::*mcherry]* (left panels) and schematic representations of the corresponding four cells (right panels) in wild-type (H) and *ham-1(gm279)* mutant (I) larvae. The Q.p-derived neurons PVM and SDQL are also labelled by persistent mCherry from *rdvIs1*. Scale bars: 10 μm. (J) V5.paap/V5.paaa cell size ratios in wild-type and *ham-1* mutant larvae.

*ham-1* mutants display two phenotypes: a loss of DCSA and a coincident production of extra neurons, presumably the result of a failure to execute the apoptotic fate. One possible explanation for these phenotypes is that the primary function of *ham-1* is to impose DCSA, and this function increases the probability that the smaller daughter will die. This latter hypothesis predicts that HAM-1 regulates DCSA in divisions that do not produce apoptotic cells, or a(S)P(S)-type divisions. To test this hypothesis, we first looked at the T.pp larval neuroblast ACD that generates the smaller anterior T.ppa daughter that differentiates into the PVW interneuron and the larger posterior T.ppp mitotic cell [31]. Using a membrane-bound GFP marker expressed in the V and T lineages, we measured the T.ppp to T.ppa wild-type cell size ratio and observed that the posterior daughter is 2 to 3 times larger than its anterior sister (Fig 3A–3C and 3E). DCSA was reduced or lost in the *ham-1(gm279)* null mutant (Fig 3D and 3E).

We next turned to the V5.pa lineage that produces cells of the posterior deirid sensillum (Horvitz and Sulston). Fluorescent markers expressed from the *egl-17* promoter label the cells of the V5.pa lineage and allowed us to directly observe the size of daughter cells at each ACD (S1 Fig, Fig 3F–3J). Two divisions in the V5.pa lineage generated daughters of unequal sizes (S1 Fig). V5.paap is the last cell to divide and generates the larger anterior V5.paapa daughter cell, which differentiates as the PVD touch neuron, and the smaller posterior V5.paapp daughter cell, which dies. Neither DCSA nor V5.paapp apoptosis was defective in *ham-1* mutants (data not shown). We noticed that the V5.paa neuroblast division generarated two surviving daughters of different size: the anterior V5.paaa daughter cell, which differentiates into the PDE neuron, was on average half the size of the posterior sister V5.paap (Fig 3G, 3H and 3J). This size asymmetry was either lost or reversed in *ham-1* mutants (Fig 3I and 3J). Although the resulting PDE neurons were born larger, and the PVD neurons ended up smaller after V5.paap divided, no obvious terminal phenotype was associated with those *ham-1* defects. One interesting phenotype is the production of extra PDE neurons in *ham-1* mutants [28]. Our analysis of this lineage does not reveal how *ham-1* mutants generate these extra cells as no extra cells were produced. Perhaps a cell in the V5.pa lineage requires *ham-1* to differentiate properly, and may aberrantly adopt the PDE fate in *ham-1* mutants.

Using Nomarski optics, Sulston and Horvitz observed that each of the larval hypodermal P cells divide to produce an anterior Pn.a cell with a larger nucleus and a posterior Pn.p cell with a smaller nucleus, suggesting that these are A(S)p(S) divisions [1]. Expression of GFP from the *egl-17* promoter in *gmIs81* transgenic animals labels the lineage of six of these cells, P3-8, and allowed us to confirm that the P3-8.a cells are larger that P3-8.p cells (ratio a-p = 1.8±0.3; n = 35). Our model predicts that these A(S)p(S) divisions should be unaffected by *ham-1* loss, and we found that *ham-1(gm279)* mutant P3-8.a cells are also larger than P3-8.p cells (ratio a-p = 1.8±0.4; n = 31). There is no significant difference between Pn daughter cells size ratios in wild-type and *ham-1* mutant animals. Taken together, our observations suggest that *ham-1* is a general regulator of divisions that produce smaller anterior and larger posterior cells independent of whether those divisions produce a daughter cell fated to die.

## Discussion

Metazoans use apoptosis to discard abnormal cells, control cell number, and shape the development of organs. In the nematode *C*. *elegans*, many ACDs that result in DCSA produce apoptotic cells. Screens for mutants with apoptosis defects has identified several genes required for DCSA, but how the molecules that they encode regulate DCSA remains unclear.

From our extensive survey of *ham-1* mutant defects, a pattern emerges where *ham-1* loss results in the production of extra cells in divisions that normally produce an apoptotic daughter cell. There are several ways to explain the extra cell phenotype: 1) transformation of the apoptopic daughter cell into its sister; 2) increasing the mitotic potential of the cell that normally survives to generate more cells; 3) the production of the extra neuroblasts earlier in the lineage; or 4) the neuroblast-like transformation of cells outside of the lineage. Markers used to identify the HSN/PHB and Q.a neuroblasts are inconsistent with the latter two possibilities because we do not observe extra neuroblasts for these lineages (unpublished observations). We favor the hypothesis that the extra neurons result from the transformation of the apoptotic cell into its sister. In support of this hypothesis, imaging of the Q.a division revealed that both cells survive and differentiate in *ham-1* mutants (unpublished observations). These observations, however, do not rule out the possibility that *ham-1* mutants generate the extra cells in other ways.

Our survey also suggests that the affected divisions in *ham-1* mutants are primarily those that produce smaller anterior and larger posterior daughter cells. Two types of findings support this hypothesis. First, *ham-1* mutations altered DCSA in all of the divisions of this type that we analyzed: the HSN/PHB divisions, the Q.a divisions, the T.pp divisions, the V5.paa divisions and the six divisions that we analyzed in the early embryo. *ham-1* mutations, by contrast, generally did not alter divisions that produce a larger anterior and smaller posterior daughter cell: the Q.p divisions, the V5.paap divisions, the six P3-8 divisions and five of the six embryonic divisions that we followed. Second, there is a correlation between lineages that produce a smaller anterior cell that dies and the production of extra cells in *ham-1* mutants. These observations are indirect and hence, a less compelling argument in favor of the model than directly observing the divisions. We note, however, that in all of the divisions that produce extra cells and that we followed, the asymmetry of the division was altered.

If the extra cells in *ham-1* mutants result from the survival of a cell normally fated to die, DCSA may contribute to the apoptotic fate of the smaller daughter cell by regulating the levels of caspase activity in the two daughter cells [10]. By quantifying caspase activity using a fluorescent caspase substrate at the centrosomes, Chakraborty et al found higher caspase levels on the side of the NSM neuroblast that will produce the smaller apoptotic cell [32]. One possibility is that caspase activity is graded in ACDs that produce apoptotic cells and that DCSA ensures that the smaller cells will have sufficient levels of active caspase to promote apoptosis. In this model, loss of DCSA would dilute caspase activity, either slowing down apoptosis execution or lowering it below a threshold required for apoptosis [10].

Neuronal differentiation also requires HAM-1. Loss of HAM-1, for example, leads to HSN and PHB neurons differentiation defects: HSNs often fail to migrate, PHB dendrites can be defective, and both cells occasionally fail to express differentiation markers [14, 33]. HAM-1 localizes asymmetrically in the HSN/PHB neuroblast, which divides to produce a cell that dies and the precursor to the HSN and PHB neurons [2, 14]. If HAM-1 is solely involved in regulating the division plane of the HSN/PHB neuroblast, perhaps the levels of asymmetrically distributed determinants that contribute to HSN and PHB fate are altered in the mutant.

Our study also shows that HAM-1 regulates DCSA in divisions that produce two daughter cells that survive. The role, if any, of daughter cell size in these lineages is unknown. The *ham-1* mutant V5.pa lineage does produce an extra PDE-like neuron [28], although the source of this extra neuron is not known. DCSA is prevalent in many divisions that occur during metazoan development, but its role in daughter cell function is often obscure. A recent study showed that DCSA coordinated the fates of daughter cells to promote the proper migration of endothelial cells during zebrafish angiogenesis [34].

Our results support a model where *ham-1*-independent divisions produce DCSA with a reversed polarity. Time-lapse imaging of the Q.a and Q.p divisions previously revealed the existence of two distinct mechanisms to generate DCSA [9]. The Q.a neuroblast divides by a spindle-independent, myosin II-dependent mechanism to establish the a(x)P(S)-type asymmetry. By contrast, the Q.p neuroblast divides by a spindle-dependent, myosin independent mechanism to establish the opposite polarity A(S)p(x), and *ham-1* is required for the Q.a but not the Q.p division [9, 13]. One possibility is HAM-1 regulates spindle-independent, myosin-dependent asymmetric divisions. Further investigations will be needed to test this hypothesis.

Both Q.a and Q.p DCSA require the function of the genes *pig-1*, *cnt-2* and *grp-1*, indicating that these neuroblasts also use overlapping mechanisms to generate different sized daughter cells [6, 9, 11, 12]. Q.a but not Q.p, by contrast, requires the genes *ham-1* and *toe-2* for DCSA [13, 22]. TOE-2 is a DEP-domain containing protein that was originally shown to be a target of MAP kinase that acts as an anti-apoptotic factor in *C*. *elegans* meiotic cells [35]. TOE-2 dynamically localizes to the cortex, the nucleus, and, like PIG-1, to the centrosomes [22]. Loss of TOE-2 cortical localization correlates with the loss of Q.a DCSA function [22]. Similar to TOE-2, HAM-1 also localizes to the cortex and nucleus and has been reported to act as a transcription factor that regulates the DCSA gene *pig-1* [14, 15, 24]. In addition to DCSA regulation, TOE-2 specifies posterior cell fates in in the Q and Q.p divisions, a function that is distinct from its role in DCSA [22]. The Wnt pathway in *C*. *elegans* specifies the fate of posterior daughter cells in many cell divisions, raising the interesting possibility that Wnt genes might coregulate posterior cell fates and organize DCSA along the AP axis in cooperation with DCSA regulators like TOE-2 [36]. Further work will be aimed at how these various molecules act together to establish DCSA.

## Supporting information

S1 Fig Cell divisions and unequal cytoplasmic partitioning in the V5.pa lineage. (A) Schematic diagram of the V5.pa lineage, which produces two neurons (PDE and PVD), two neuron-associated support cells (PDE socket cell and PDE sheath cell) and one apoptotic cell (V5.paapp). V5.paa and V5.paap daughters are unequal in size, whereas V5.pap division is symmetric in size. (B) Description of the cell division sequence and cell movements that occur during posterior deirid development. Left panels are representative fluorescent labeling of the left V5.pa lineage at all stages of development by *rdvIs1[egl-17p*::*myristoylated mcherry; egl-17p*::*H2B*::*mcherry]*. The cells boundaries are indicated by dotted lines and the name of each cell is indicated. Right panels are schematic representations of the corresponding cells. Black arrows describe cell displacements. Colored arrows indicate the direction of neurite extensions. Upon V5.pa division, the anterior daughter V5.paa moves to a more dorsal position above its sister V5.pap. The V5.paa neuroblast divides asymmetrically before V5.pap to produce the smaller anterior daughter V5.paaa, which will become the PDE neuron, and the larger posterior daughter V5.paap. The V5.pap glioblast's division produces daughter cells of equal size, the anterior daughter V5.papa, which will become the PDE socket cell, and the posterior daughter V5.papp, which will become the PDE sheath cell. Before the V5.paap division, both V5.paap and V5.paaa migrate anteriorly and ventrally, respectively. V5.paap then divides to generate the larger anterior daughter V5.paapa which will become the PVD neuron, and the smaller posterior daughter V5.paapp, which dies. After completion of all cell divisions, PDE and PVD neurons extend their neurites. Abbreviations: so: socket cell; sh: sheath cell. Scale bars: 10 μm.
(TIF)
