# Supplementary figures and images for: The Caenorhabditis elegans gene ham-1 regulates daughter cell size asymmetry primarily in divisions that produce a small anterior daughter cell
Source: PLoS One. 2018 Apr 18;13(4):e0195855. doi: 10.1371/journal.pone.0195855 (PMC5905977; doi:10.1371/journal.pone.0195855)

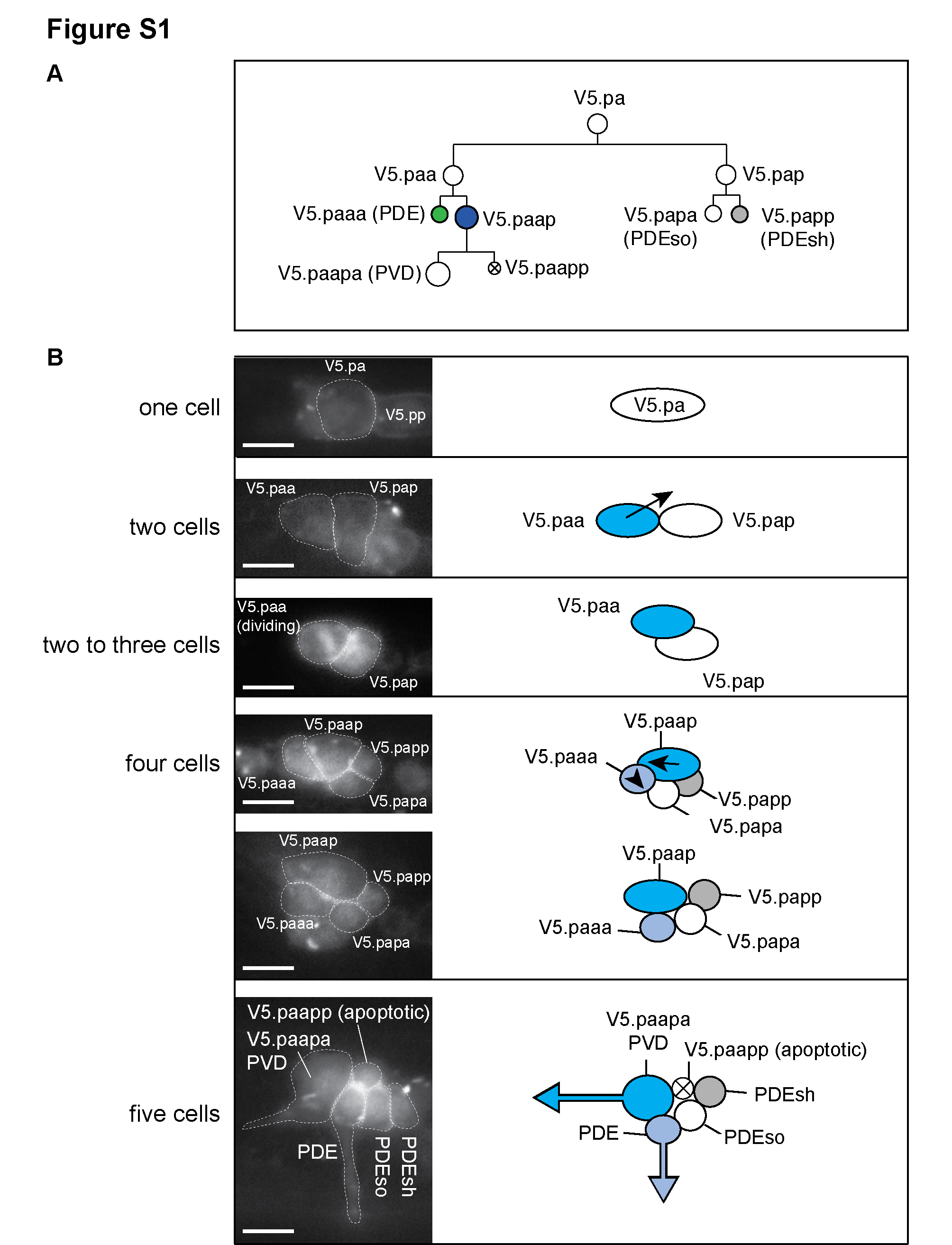

Supplement: S1 Fig — (A) Schematic diagram of the V5.pa lineage, which produces two neurons (PDE and PVD), two neuron-associated support cells (PDE socket cell and PDE sheath cell) and one apoptotic cell (V5.paapp). V5.paa and V5.paap daughters are unequal in size, whereas V5.pap division is symmetric in size. (B) Description of the cell division sequence and cell movements that occur during posterior deirid development. Left panels are representative fluorescent labeling of the left V5.pa lineage at all stages of development by rdvIs1[egl-17p::myristoylated mcherry; egl-17p::H2B::mcherry]. The cells boundaries are indicated by dotted lines and the name of each cell is indicated. Right panels are schematic representations of the corresponding cells. Black arrows describe cell displacements. Colored arrows indicate the direction of neurite extensions. Upon V5.pa division, the anterior daughter V5.paa moves to a more dorsal position above its sister V5.pap. The V5.paa neuroblast divides asymmetrically before V5.pap to produce the smaller anterior daughter V5.paaa, which will become the PDE neuron, and the larger posterior daughter V5.paap. The V5.pap glioblast's division produces daughter cells of equal size, the anterior daughter V5.papa, which will become the PDE socket cell, and the posterior daughter V5.papp, which will become the PDE sheath cell. Before the V5.paap division, both V5.paap and V5.paaa migrate anteriorly and ventrally, respectively. V5.paap then divides to generate the larger anterior daughter V5.paapa which will become the PVD neuron, and the smaller posterior daughter V5.paapp, which dies. After completion of all cell divisions, PDE and PVD neurons extend their neurites. Abbreviations: so: socket cell; sh: sheath cell. Scale bars: 10 μm. (TIF) [file pone.0195855.s001.tif]
